# KYNA Ameliorates Glutamate Toxicity of HAND by Enhancing Glutamate Uptake in A2 Astrocytes

**DOI:** 10.3390/ijms25084286

**Published:** 2024-04-12

**Authors:** Jie Chen, Jinhu Zou, Pengwei Huang, Xuefeng Gao, Jingxian Lun, Yubin Li, Zelong Gong, Hong Cao

**Affiliations:** Department of Microbiology, School of Public Health, Southern Medical University (Guangdong Provincial Key Laboratory of Tropical Disease Research), Guangzhou 510515, China; chen1617538946@outlook.com (J.C.);

**Keywords:** kynurenic acid, HIV-1 gp120, A2 astrocyte, glutamate, α7nAChR, EAAT2, NF-κB

## Abstract

Reactive astrocytes are key players in HIV-associated neurocognitive disorders (HAND), and different types of reactive astrocytes play opposing roles in the neuropathologic progression of HAND. A recent study by our group found that gp120 mediates A1 astrocytes (neurotoxicity), which secrete proinflammatory factors and promote HAND disease progression. Here, by comparing the expression of A2 astrocyte (neuroprotective) markers in the brains of gp120 tgm mice and gp120^+^/α7nAChR^−/−^ mice, we found that inhibition of alpha 7 nicotinic acetylcholine receptor (α7nAChR) promotes A2 astrocyte generation. Notably, kynurenine acid (KYNA) is an antagonist of α7nAChR, and is able to promote the formation of A2 astrocytes, the secretion of neurotrophic factors, and the enhancement of glutamate uptake through blocking the activation of α7nAChR/NF-κB signaling. In addition, learning, memory and mood disorders were significantly improved in gp120 tgm mice by intraperitoneal injection of kynurenine (KYN) and probenecid (PROB). Meanwhile, the number of A2 astrocytes in the mouse brain was significantly increased and glutamate toxicity was reduced. Taken together, KYNA was able to promote A2 astrocyte production and neurotrophic factor secretion, reduce glutamate toxicity, and ameliorate gp120-induced neuropathological deficits. These findings contribute to our understanding of the role that reactive astrocytes play in the development of HAND pathology and provide new evidence for the treatment of HAND via the tryptophan pathway.

## 1. Introduction

With the spread of antiretroviral therapy, HAND has become the most prevalent neurological complication in 39 million HIV-infected patients worldwide, manifesting as unresponsiveness, learning and memory deficits, and creating a significant burden on patients’ daily lives and socioeconomics [1,2]. HIV-1 gp120 protein, as one of the subunits of the HIV-1 envelope glycoprotein, is the first to bind to the cell surface receptor, assisting HIV-1 to enter the host cells and establish infection [3]. Our team’s previous study found that gp120 induces glial cell NLRP3-dependent cellular pyroptosis and IL-1β production, resulting in neuroinflammation and neuronal damage [4]. Astrocytes, as the most abundant glial cells in the CNS, are responsible for maintaining CNS microenvironmental homeostasis, such as the uptake and conversion of the neurotransmitter glutamate [5]. In previous studies, astrocytes usually serve as an important viral reservoir for HIV-1 in the brain, but our group’s latest study demonstrated that gp120 activates astrocyte reactivity, induces their transformation into deleterious (A1 astrocytes) and promotes the secretion of pro-inflammatory cytokines [6]. Furthermore, under normal physiological conditions, astrocytes maintain glutamate homeostasis by taking up the vast majority of free glutamate released from neuronal synapses via glutamate transporter proteins (EAATs) [7]. However, glutamate toxicity is one of the triggers for neurodegenerative diseases, such as HAND and AD [8,9]. HIV-1 gp120 may therefore impair the glutamate uptake capacity of astrocytes, thereby mediating the occurrence of glutamate toxicity in HAND, but the specific mechanism by which gp120 impairs the glutamate uptake capacity of astrocytes has not yet been reported.

As an endogenous metabolite of astrocyte KP, KYNA exerts anti-inflammatory and antioxidant functions by binding to aryl hydrocarbon receptor (AHR) and is widely used in treating neurodegenerative diseases [10,11,12]. Notably, KYNA reduced excitotoxicity by blocking α7nAChR, NMDAR and GPR35 in neurons [12,13,14,15]. In addition, our group recently found that the brain endogenous metabolite KYNA inhibits A1 astrocyte formation in the gp120 mouse brain [6]. Critically, studies have shown that astrocytes can activate into two polarized states: a neurotoxic or inflammatory phenotype (A1) and a neuroprotective or anti-inflammatory phenotype (A2) [16]. Notably, quinolinic acid (QUIN), one of the end products of tryptophan metabolism, was shown to not only inhibit glutamate uptake by astrocytes, but also to be able to cause neurological damage [17,18]. Therefore, KYNA can reduce glutamate toxicity of HAND by improving the glutamate uptake capacity of A2 astrocytes. Meanwhile, it promotes A2 astrocytes to generate increased neurotrophic factor levels, reduce neuronal damage and increase synaptic plasticity. In summary, we propose a hypothesis that KYNA ameliorates neuronal injury in HAND by enhancing the number and glutamate uptake capacity of A2 astrocytes.

## 2. Results

### 2.1. HIV-1 gp120 Inhibits A2 Astrocyte Formation and Induces Glutamatergic Toxicity

**Figure 1:**
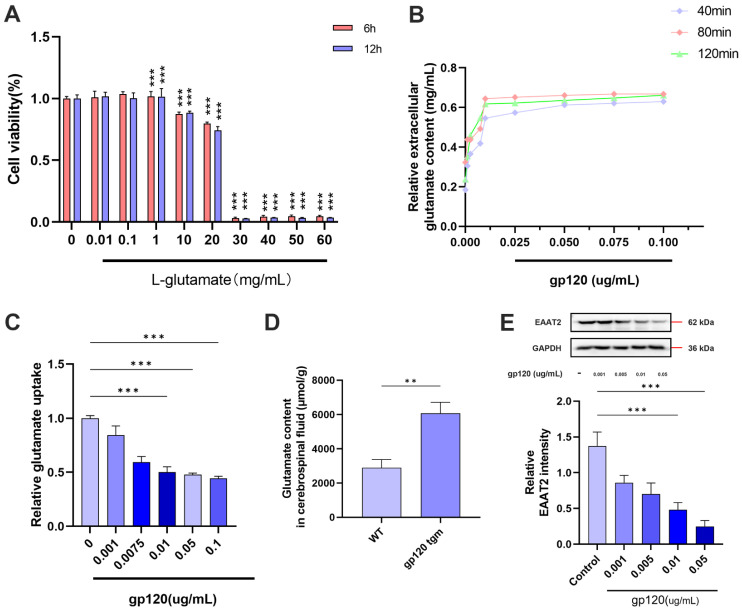
The effect of HIV-1 gp120 on the glutamate uptake capacity of primary astrocytes. (**A**) Viability of primary astrocytes by CCK8 measurement of incremental concentrations of L-glutamate. (**B**) Glutamate concentration in the supernatant of gp120-treated primary astrocytes. (**C**) Influence of gp120 on glutamate uptake capacity of primary astrocytes. (**D**) Glutamate content in the cerebrospinal fluid of WT mice and gp120 tgm mice. (**E**) gp120 inhibits EAAT2 protein expression in primary astrocytes. ** *p* < 0.01, and *** *p* < 0.001.

Studies have shown that glutamate toxicity is a key pathogenic mechanism of HAND, and that the glutamate transporter protein 2 (EAAT2) of astrocytes takes up most of the glutamate released from neuronal synapses [19,20]. To investigate the effect of gp120 on glutamate content in the CNS, we analyzed the glutamate content in the cerebrospinal fluid of WT and gp120 tgm mice and found that the glutamate content in the cerebrospinal fluid of gp120 tgm mice was significantly higher than that of WT mice (Figure 1D). To further understand the effect of gp120 on the glutamate uptake capacity of reactive astrocytes, we first treated primary astrocytes with different concentrations of glutamate and found that 1 mg/mL of glutamate had no effect on astrocyte viability (Figure 1A). Next, we treated primary astrocytes with different concentrations of gp120 protein for 24 h, and then incubated the cells with PBS solution containing 1 mg/mL glutamate. Finally, the glutamate content in PBS was detected at 40 min, 80 min and 120 min, respectively. The results showed that the extracellular glutamate content increased with increasing gp120 concentration, indicating that the glutamate uptake capacity of reactive astrocytes decreased with increasing gp120 concentration (Figure 1B,C). Western blot results showed that the astrocyte EAAT2 protein content decreased with increasing gp120 concentration (Figure 1E), indicating that gp120 was able to inhibit the expression of EAAT2 in astrocytes. And 0.01 μg/mL of gp120 was the optimal concentration for constructing a vitro glutamate toxicity cell model.

**Figure 2:**
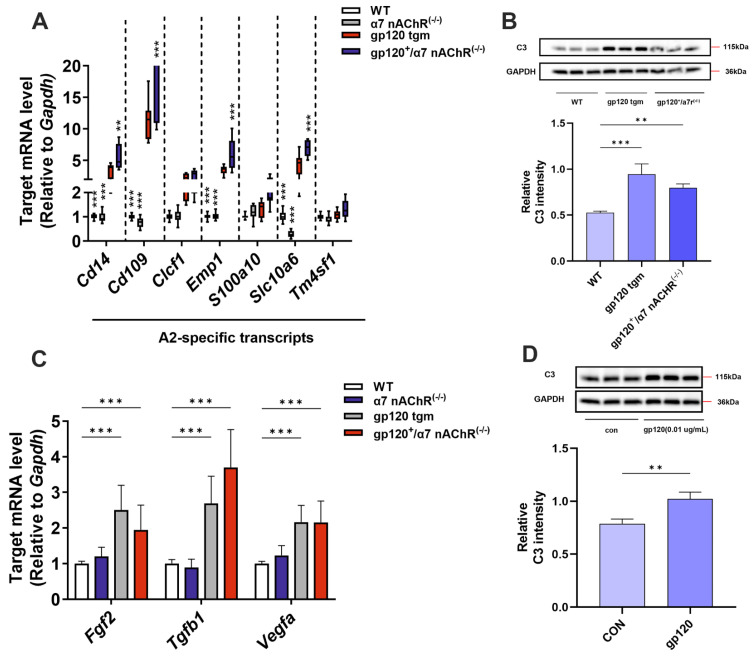
The effect of HIV−1 gp120 on mouse brain A2 astrocytes. Spectrum of mRNA expression of A2−specific genes (**A**) and trophic factors (**C**) in mouse brain parenchyma. (**B**) C3 protein levels in brain parenchyma of WT mice and gp120 tgm mice. (**D**) Expression levels of C3 protein in gp120−treated primary astrocytes. ** *p* < 0.01, and *** *p* < 0.001.

Our team’s latest research shows that gp120 is able to induce A1 astrocyte formation [6]. However, it is recognized that there are two states of reactive astrocytes, A1 (neurotoxic) and A2 (neuroprotective) [16]. Therefore, we next verified both in vivo and in vitro whether gp120 could inhibit A2 astrocyte formation. We found that the expression of specific genes for A2 astrocytes and neurotrophic factor genes was down-regulated in the brain parenchyma of gp120 tgm mice compared with that of WT mice (Figure 2A,C). And we found that the C3 protein level was elevated in gp120 tgm mice compared with WT mice by examining the expression level of the A1 astrocyte-specific marker C3 (Figure 2B). In addition, gp120 was able to increase the level of C3 expression in primary astrocytes (Figure 2D). These results suggest that gp120 inhibits A2 astrocyte formation. In summary, gp120 was able to inhibit the expression of A2 astrocyte EAAT2 and induce glutamate toxicity.

### 2.2. KYNA Promotes A2 Astrocyte Formation and Reduces gp120-Induced Glutamate Toxicity

**Figure 3:**
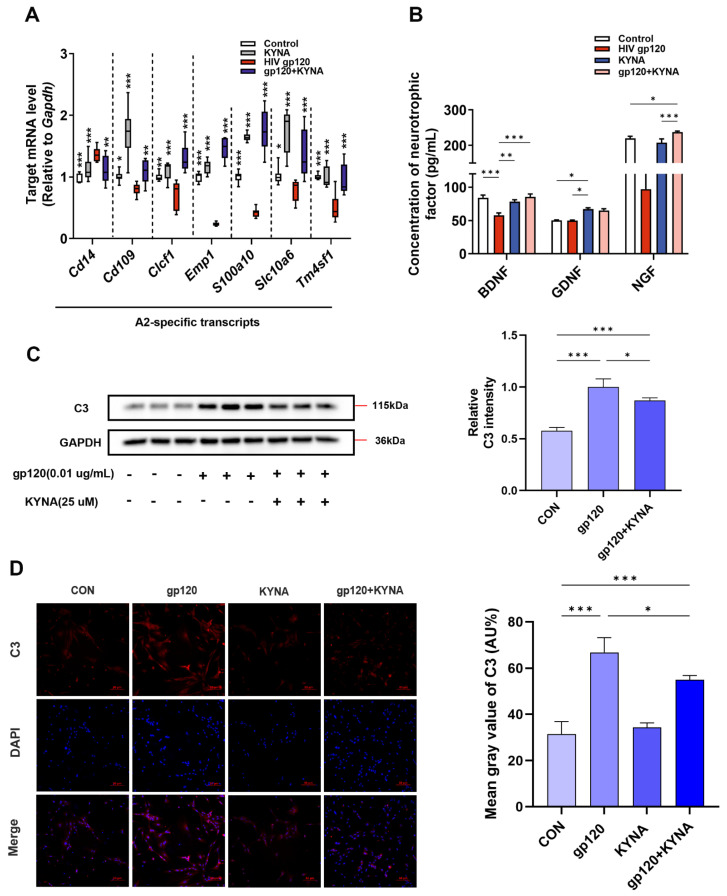
KYNA promotes A2 astrocyte formation. (**A**) The mRNA expression profiles of A2−specific genes in KYNA and gp120−treated primary astrocytes. (**B**) Trophic factor content in the supernatant of primary astrocytes. (**C**) Quantification of C3 protein levels in primary astrocytes by Western blot. (**D**) Immunofluorescence images showing the expression of C3 in primary astrocytes (scale bar: 50 μm), and quantified using ImageJ software (ImageJ 1.54f). * *p* < 0.05, ** *p* < 0.01, and *** *p* < 0.001.

KYNA has neurotransmitter modulating, antioxidant and anti-inflammatory features that can improve learning and memory deficits in neurodegenerative diseases [21,22]. And our group’s previous study showed that KYNA inhibited A1 astrocyte activation, so we next needed to verify whether KYNA could promote A2 astrocyte generation. First, we treated primary astrocytes with 25 μM KYNA alone or in combination with gp120, and RT-qPCR results showed that KYNA was able to up-regulate A2 astrocyte-specific genes suppressed by gp120 (Figure 3A). Meanwhile, KYNA could down-regulate the expression of C3 protein, a type A1-specific marker, compared with the gp120 alone treatment group (Figure 3C,D). In addition, we examined the neurotrophic factor levels in cell supernatants, and the ELISA results showed that KYNA could up-regulate the secretion of neurotrophic factor in A2 astrocytes (Figure 3B). These data demonstrated that KYNA was able to promote the formation of A2 astrocytes.

**Figure 4:**
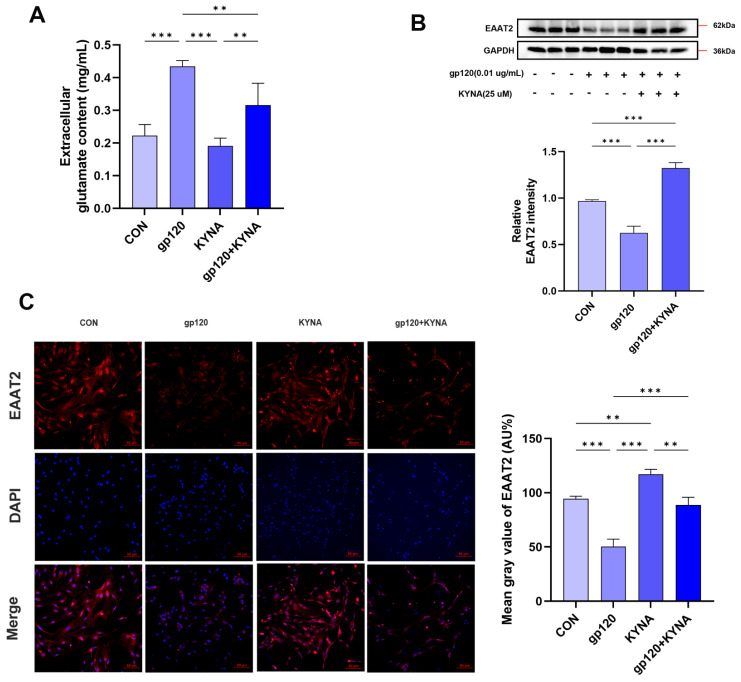
Effect of KYNA on glutamate uptake capacity of primary astrocytes. (**A**) Glutamate content in the supernatant of KYNA−treated primary astrocytes. (**B**) Expression of EAAT2 in KYNA and gp120−treated primary astrocytes was assessed by Western blot. (**C**) Immunofluorescence images showing the expression of EAAT2 in primary astrocytes (scale bar: 50 μm), and quantitatively analyzed using ImageJ software (ImageJ 1.54f). ** *p* < 0.01, and ***p < 0.001.

Studies have shown that the astrocyte endogenous metabolite KYNA modulates extracellular glutamate levels in rat prefrontal cortex and hippocampus [23,24]. To explore the effect of KYNA on the glutamate uptake capacity of reactive astrocytes, we first verified that KYNA significantly reduced the glutamate content in cell supernatants by glutamate measurement experiments (Figure 4A), suggesting that KYNA significantly improved the glutamate uptake capacity of astrocytes damaged by gp120. To further investigate this issue, we used immunofluorescence and Western blot, respectively. As shown in Figure 4B,C, KYNA significantly up-regulated the expression of EAAT2 in A2 astrocytes compared to the gp120-treated group. Overall, these data suggest that KYNA enhances the ability of astrocytes to protect neurons from glutamate toxicity by up-regulating the expression of EAAT2 in A2 astrocytes.

### 2.3. KYNA Enhances Glutamate Uptake in A2 Astrocytes by Blocking α7nAChR

**Figure 5:**
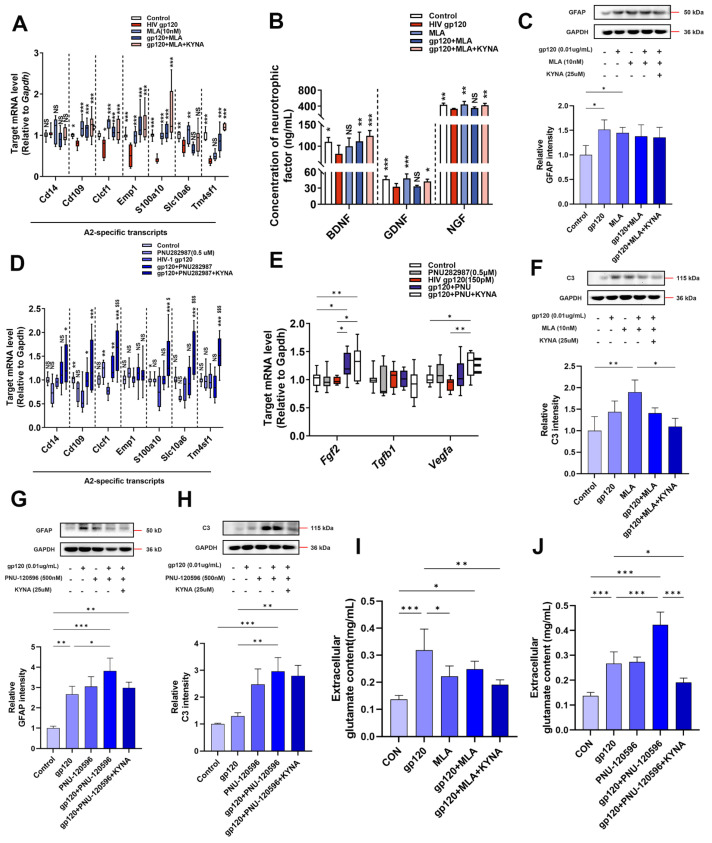
Effects of α7nAChR antagonist (MLA) and agonist (PNU−282987) on A2 astrocyte formation. (**A**,**D**) The mRNA expression profiles of A2−specific genes in the MLA− and PNU−282987−treated primary astrocytes. (**B**) Trophic factor content in the supernatant of primary astrocytes. Expression levels of GFAP (**C**,**G**) and C3 (**F**,**H**) in MLA− and PNU−282987−treated primary astrocytes. (**E**) mRNA expression profiles of trophic factors in PNU−282987−treated A2 astrocytes. (**I**,**J**) Glutamate content in the supernatant of MLA and PNU−282987−treated primary astrocytes. * represents comparison with control group. NS means *p* > 0.05, * *p* < 0.05, ** *p* < 0.01, and *** *p* < 0.001. $ represents comparison with HIV-1 gp120 group. $ represents *p* < 0.05, $$$ represents *p* < 0.001.

Under normal physiological conditions, endogenous KYNA acts preferentially as a negative allosteric regulator of α7nAChR [25]. To verify whether α7nAChR is involved in regulating A2 astrocytes, we utilized a gp120 tgm mouse model with a null mutation in the α7nAChR gene (gp120^+^/α7nAChR^−/−^ mouse). The results showed that A2 astrocyte marker genes and neurotrophic factor genes were higher in gp120^+^/α7nAChR^−/−^ mice than gp120 tgm mice (Figure 2A,C), which preliminarily suggests that blockade of α7nAChR can promote A2 astrocyte formation. To further explore the specific role of α7nAChR, we treated primary astrocytes using MLA (specific α7nAChR antagonist) and PNU-282987 (specific α7nAChR agonist), respectively. Figure 5C,G shows that GFAP expression was up-regulated in each treatment group compared to the control group, saying that the star glial cells were more responsive. In addition, the KYNA+MLA group was able to further up-regulate the expression of the A2 signature gene and reduce the content of the A1 marker C3 protein (Figure 5A,F). Meanwhile, ELISA results showed that the KYNA+MLA group was able to up-regulate the neurotrophic factor content in the astrocyte supernatant compared with the gp120 group (Figure 5B). However, the KYNA+PNU-282987 group produced opposite results on A2 markers and neurotrophic factors (Figure 5D,E,H). These results consistently suggest that blocking α7nAChR promotes A2 astroglia formation.

**Figure 6:**
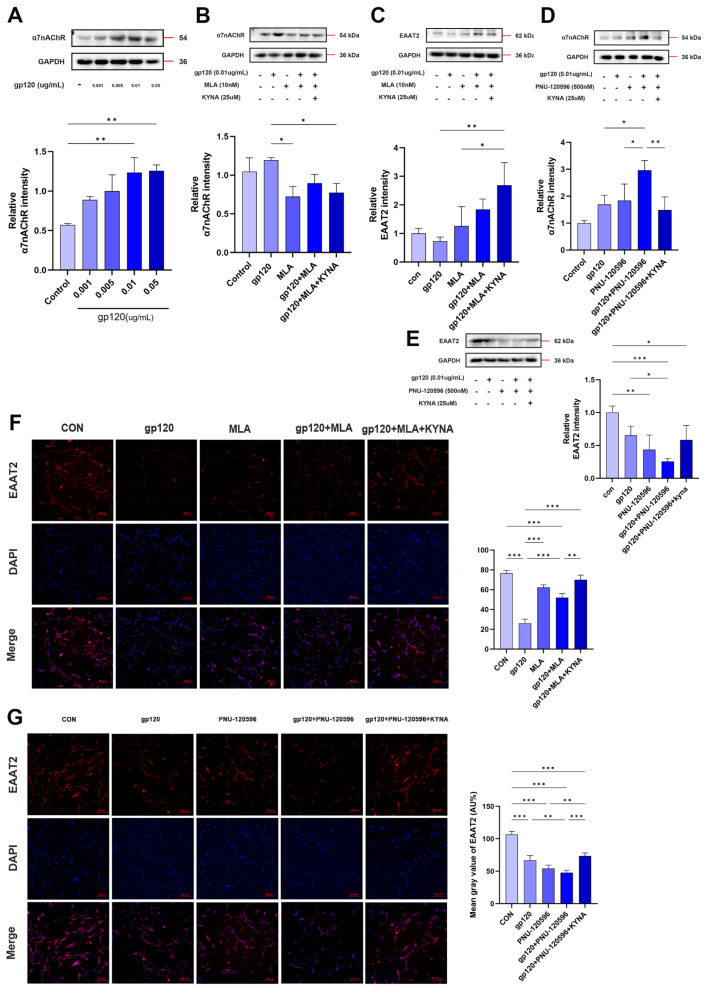
The effects of α7nAChR antagonist (MLA) and agonist (PNU−282987) on the glutamate uptake capacity of A2 astrocytes. (**A**) Levels of α7nAChR in gp120−treated primary astrocytes. The levels of MLA− and PNU−282987−treated primary astrocytes α7nAChR proteins (**B**,**D**), and EAAT2 proteins (**C**,**E**). Immunofluorescence showed the expression of EAAT2 protein in MLA− (**F**) and PNU−282987− (**G**) treated primary astrocytes (scale bar: 50 μm), and quantitative analysis was performed using ImageJ software (ImageJ 1.54f). * *p* < 0.05, ** *p* < 0.01, and *** *p* < 0.001.

Next, we explored the effect of α7nAChR on the glutamate uptake capacity of astrocytes. First, we explored the effect of gp120 on α7nAChR in astrocytes by WB experiments. As shown in Figure 6A, the α7nAChR content was positively proportional with gp120 concentration. Then, we detected the glutamate content in the supernatant of astrocytes pretreated with MLA and PNU-282987 for 1h by glutamate content assay experiment. As shown in Figure 5I, the glutamate content in the supernatant of the gp120+MLA group was significantly lower than the gp120 group and higher than the MLA group. And the glutamate content in the supernatant was further reduced after KYNA + MLA, and it is noteworthy that PNU-282987 pretreatment resulted in the opposite experimental results (Figure 5J). In addition, we verified the regulation of α7nAChR by MLA and PNU-282987 treatments, and the Western blot results in Figure 6B,D were as we expected. Finally, we detected the expression of EAAT2 in astrocytes by Western blot and immunofluorescence, and the results showed that KYNA+MLA significantly reduced the inhibitory effect of gp120 on EAAT2 (Figure 6C,F). On the other hand, PNU-282987 pretreatment enhanced the gp120 damage to EAAT2 (Figure 6E,G). From the comparison of the above experimental results utilizing α7nAChR antagonists and agonists, it is reasonable to believe that KYNA enhances EAAT2 protein expression and improves glutamate uptake in A2 astrocytes by inhibiting α7nAChR.

### 2.4. KYNA Promotes A2 Astrocyte EAAT2 Expression by Blocking α7nAChR/NF-κB Signaling Activation

**Figure 7:**
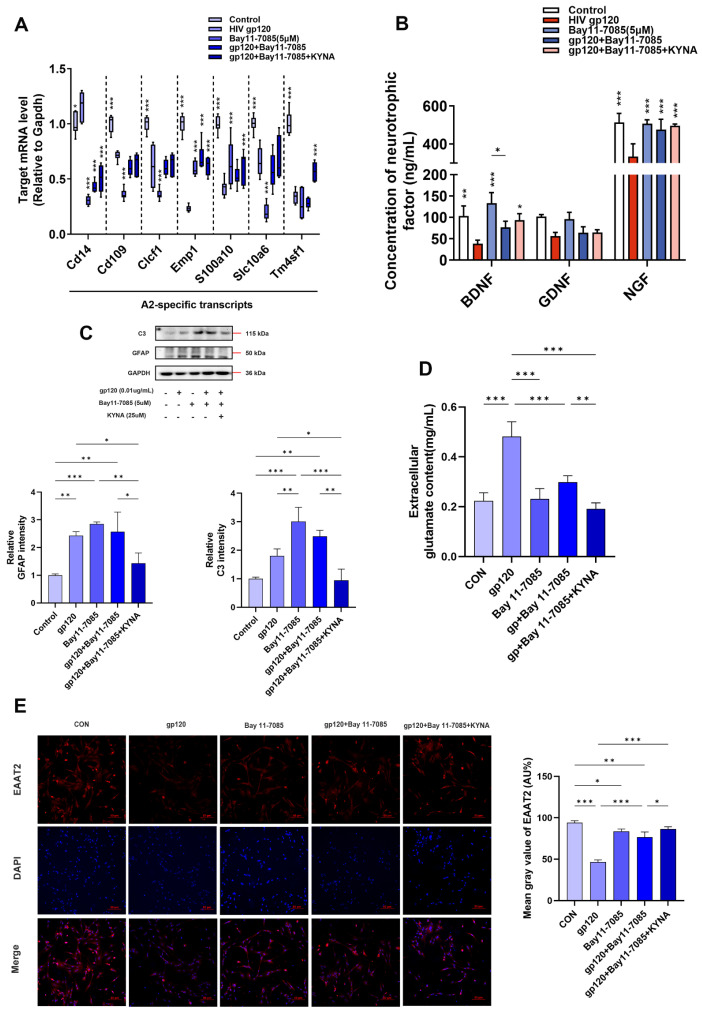
The effect of NF−κB antagonist (Bay11−7085) on A2 astrocytes. (**A**) A2−specific gene mRNA expression profile of Bay11−7085−treated primary astrocytes. (**B**) Trophic factor content in the supernatant of primary astrocytes. (**C**) Quantification of C3 and GFAP expression levels in primary astrocytes by Western blot. (**D**) Glutamate content in the supernatant of Bay11−7085−treated primary astrocytes. (**E**) Immunofluorescence images showing the expression of EAAT2 in primary astrocytes (scale bar: 10 μm). * *p* < 0.05, ** *p* < 0.01, and *** *p* < 0.001.

It has been shown that the nuclear factor-κB (NF-κB) pathway activates astrocyte responsiveness and exerts both neurotoxic and neuroprotective effects by regulating the timing and level of cytokine action [26,27]. In addition, our group’s previous studies showed that E. coli K1 mediates inflammation and meningitis by activating the α7nAChR/NF-κB signaling axis to promote bacterial crossing of the blood–brain barrier (BBB) [28,29]. Consequently, we hypothesized that the α7nAChR/NF-κB signaling axis could regulate A2 astrocyte production. To test this hypothesis, we treated primary astrocytes with Bay11-7085, an inhibitor of NF-κB activation. As shown in Figure 7A,C, astrocyte reactivity was activated compared to controls, and Bay11-7085 enhanced KYNA promotion of A2-specific gene expression and decreased expression of the A1-specific marker C3. Subsequent ELISA results confirmed that blocking NF-κB signaling on the basis of KYNA further reduced the inhibitory effect of gp120 on A2 neurotrophic factor release (Figure 7B). These data suggest that the A2 astrocyte generation can be regulated through the α7nAChR/NF-κB signaling axis.

**Figure 8:**
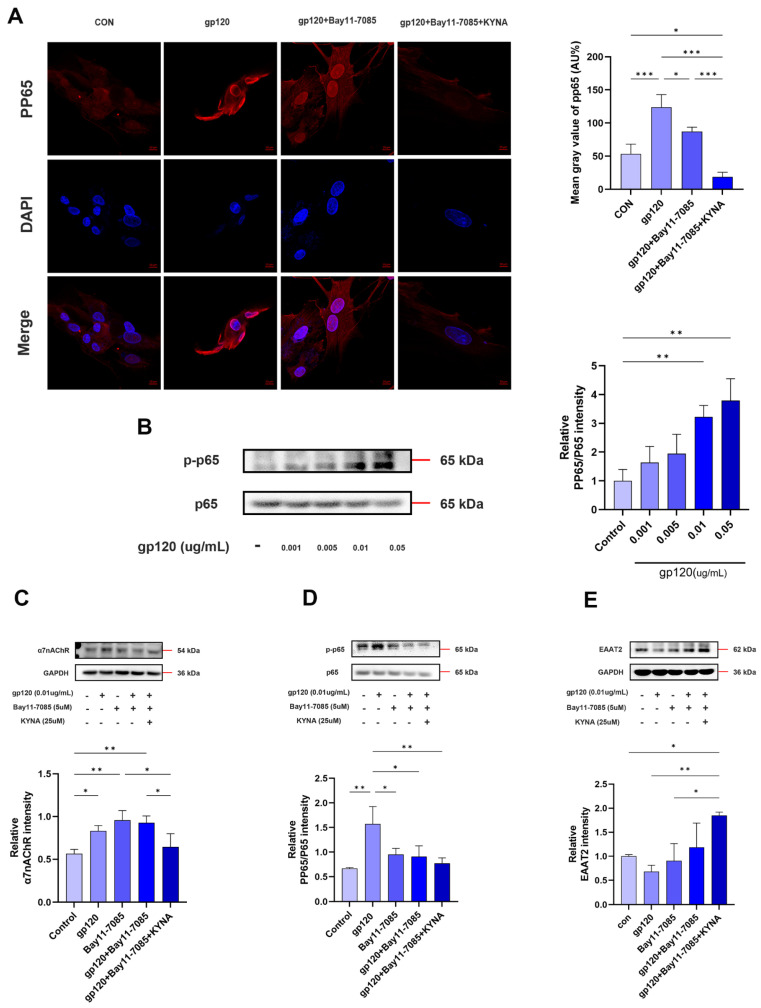
Effect of NF−κB antagonist (Bay11−7085) on glutamate uptake capacity of A2 astrocytes. (**A**) Immunofluorescence showing the expression of NF−κB subunit pp65 in primary astrocytes after Bay11−7085 treatment (scale bar: 10 μm). (**B**) Protein expression of pp65 in gp120−treated primary astrocytes. Protein levels of α7nAChR (**C**), NF−κB (**D**), and EAAT2 (**E**) were measured by Western blot in Bay11−7085−treated astrocytes. * *p* < 0.05, ** *p* < 0.01, and *** *p* < 0.001.

On the other hand, subunit p65 of NF-κB has been shown to inhibit EAAT2 expression through binding to the -583 site of the astrocyte EAAT2 promoter [30,31]. To explore the regulation of glutamate uptake capacity of astrocytes by NF-κB, glutamate assay experiments demonstrated that the use of Bay11-7085 and KYNA significantly reduced the glutamate content of cell supernatants (Figure 7D), suggesting that there is a significant improvement in the ability of astrocytes to uptake glutamate. First, we demonstrated that gp120 was able to induce NF-κB activation with a positive relationship within a certain range (Figure 8B). Next, we detected the expression of EAAT2 and NF-κB in reactive astrocytes by immunofluorescence and Western blot. The results showed that gp120-induced pp65 expression in astrocytes was reduced after Bay11-7085 and KYNA treatment (Figure 8A,D), while EAAT2 expression was enhanced (Figure 7E and Figure 8E). Interestingly, EAAT2 and NF-κB showed opposite trends under the same treatments. More importantly, KYNA together with Bay11-7085 enhanced the glutamate uptake capacity of A2 astrocytes. These results consistently suggest that KYNA promotes A2 astrocyte EAAT2 expression by inhibiting α7nAChR/NF-κB signaling activation.

### 2.5. Intraperitoneal Injection of KYN and PROB Effectively Increased KYNA Content in the Brain of gp120 tgm Mice and Reduced Neuronal Damage

**Figure 9:**
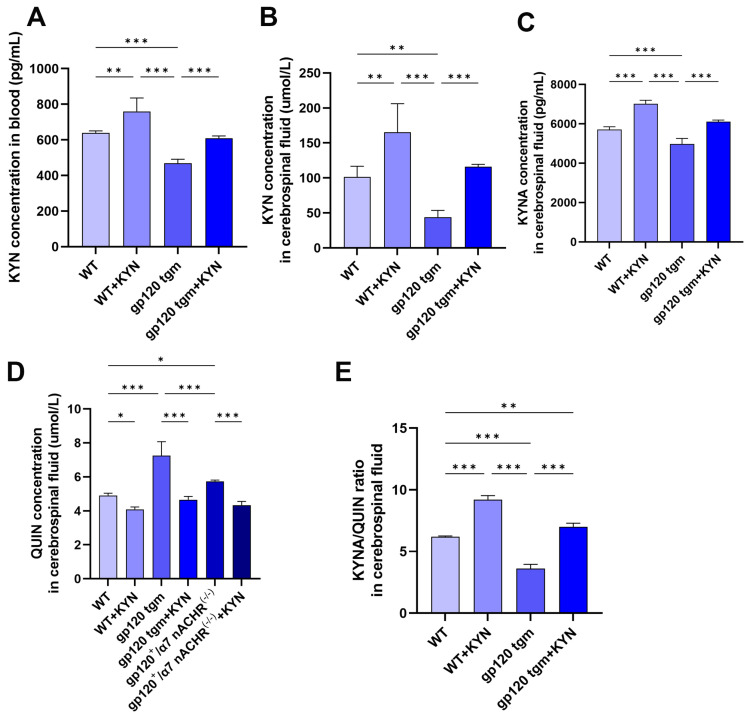
The effects of intraperitoneal injection of KYN and PROB on the metabolism of the KP pathway in mice. (**A**) KYN content in blood of WT and gp120 tgm mice. KYN (**B**), KYNA (**C**), and QUIN (**D**) levels in mouse cerebrospinal fluid were measured by ELISA. (**E**) KYNA/QUIN ratio in mouse cerebrospinal fluid. * *p* < 0.05, ** *p* < 0.01, and *** *p* < 0.001.

Our previous studies have shown that tryptophan feeding can improve neuropathology and alleviate A1 astrocyte activation in gp120 tgm mice [6]. However, the metabolism of dietary tryptophan to KYNA is a complex reaction process across the gut–brain axis, and the efficiency of KYNA conversion is influenced by factors such as the gut microbiota and the blood–brain barrier, which could not be assessed [32,33]. Therefore, this experiment used intraperitoneal injection of KYN and PROB to increase the concentration of KYNA in the mouse brain based on the tryptophan-fed mouse protocol [34]. To verify whether intraperitoneal injection of KYN and PROB was effective in increasing the KYNA content in the mouse brain, we detected the expression of KYN, KYNA and QUIN in the blood and cerebrospinal fluid of mice by ELISA. After injection of KYN and PROB, the KYN content in blood and cerebrospinal fluid of mice was significantly up-regulated, and the KYN content in gp120 tgm mice was lower than that in WT mice (Figure 9A,B). As expected, the KYNA/QUIN ratio in the cerebrospinal fluid of gp120 tgm mice was significantly lower than WT mice (Figure 9E), and gp120 tgm mice exhibited neurotoxicity of QUIN. The KYNA/QUIN ratio in the cerebrospinal fluid of gp120 tgm mice was significantly up-regulated after injection of KYN and PROB. These indicate that intraperitoneal injection of KYN and PROB can effectively increase the KYNA content in the mouse brain.

**Figure 10:**
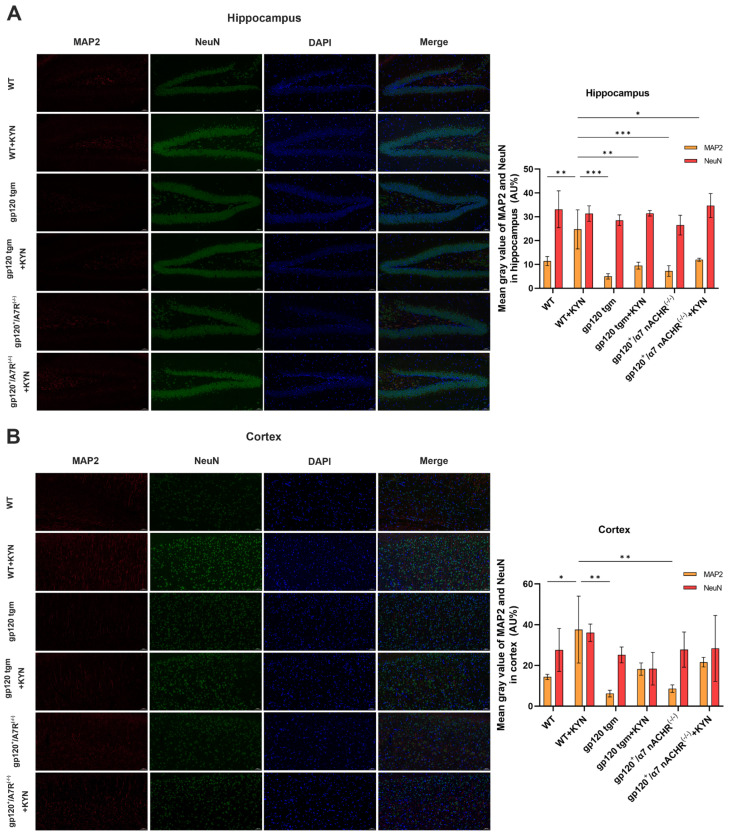
Effects of intraperitoneal injection of KYN and PROB on mouse neurons. (**A**) Immunofluorescence showing the distribution of NeuN and MAP−2 in the hippocampus (scale bar: 50 μm). (**B**) Immunofluorescence showing the distribution of NeuN and MAP−2 in the cortex (scale bar: 50 μm) and quantitative analysis using ImageJ software (ImageJ 1.54f). * *p* < 0.05, ** *p* < 0.01, and *** *p* < 0.001.

Neuronal nuclear antigen (NeuN) and microtubule-associated protein (MAP2) represent the extent of neuronal death and synaptic damage. We explored neuronal damage in mice by co-localization of NEUN (green) and MAP2 (red) immunofluorescence. As shown in Figure 10A,B, gp120 tgm mice exhibited more severe neuronal dendritic damage than WT mice in both the cortex and hippocampus, while the number of neurons did not change significantly. The neuronal dendritic damage in gp120 tgm mice was largely reversed after KYN injection. These data demonstrate that treatment with KYN and PROB ameliorates the neuropathologic defects in gp120 tgm mice.

### 2.6. Intraperitoneal Injection of KYN and PROB Ameliorates Glutamate Toxicity in gp120 tgm Mice by Promoting A2 Astrocyte Formation

**Figure 11:**
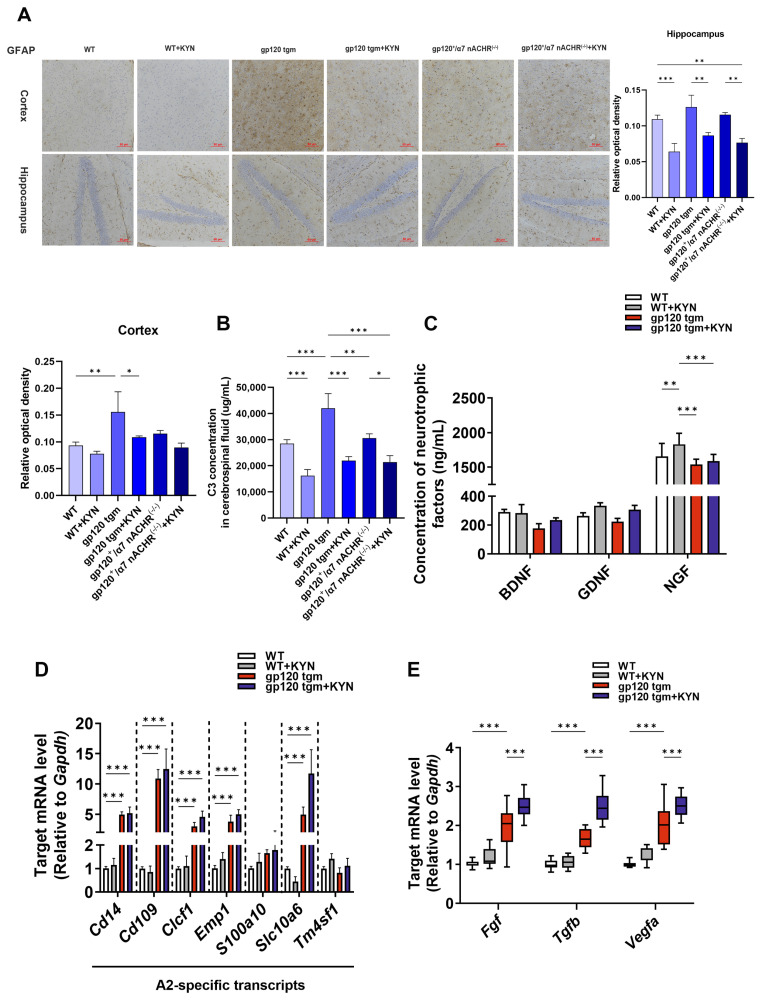
Effects of intraperitoneal injection of KYN and PROB on mouse brain A2 astrocytes. (**A**) Representative images of immunohistochemical staining of GFAP on cortical and hippocampal brain sections from gp120^+^/α7nAChR^−/−^ mice and gp120 tgm mice (scale bar: 50 μM). (**B**) A1 astrocyte marker C3 protein levels in mouse cerebrospinal fluid. (**C**) ELISA detected trophic factor levels in mouse cerebrospinal fluid. The mRNA expression profiles of A2−specific genes (**D**) and trophic factor genes (**E**) in mouse brain parenchyma. * *p* < 0.05, ** *p* < 0.01, and *** *p* < 0.001.

We hypothesized that the neuropathologic improvement in intraperitoneally injected KYN and PROB mice was correlated with A2 astrocytes. Immunohistochemical results showed that astrocyte reactivity was higher in gp120 tgm mice than in WT mice, and was significantly reduced after KYN and PROB treatment (Figure 11A). As shown in Figure 11D,E, A2 astrocyte-specific and neurotrophic factor genes were higher in the brains of gp120 tgm + KYN mice than in gp120 tgm mice, while ELISA results showed that the expression of A1 astrocyte marker C3 in the cerebrospinal fluid was lower in gp120^+^/α7nAChR^−/−^ mice than gp120 tgm mice and higher than gp120 tgm + KYN mice (Figure 11B). And the neurotrophic factor in the cerebrospinal fluid of gp120 tgm + KYN mice was higher than that of gp120 tgm mice (Figure 11C). Overall, these data suggest that intraperitoneal injection of KYN and PROB promotes the production of A2 astrocytes and secretion of neurotrophic factors in gp120 tgm mice.

**Figure 12:**
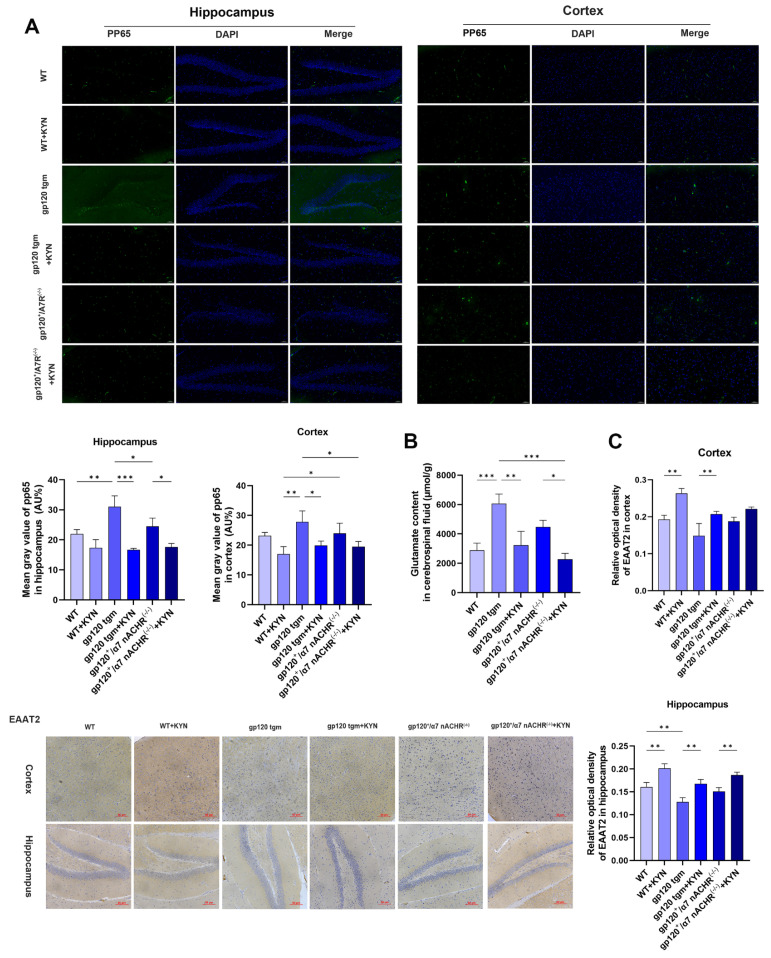
Effects of intraperitoneal injection of KYN and PROB on glutamate in mouse brain. (**A**) Immunofluorescence showing pp65 expression in the hippocampus and cortex of WT and gp120 tgm mice (scale bar: 50 μm). (**B**) Glutamate content in the brain of WT and gp120 tgm mice. (**C**) Immunohistochemistry showing the expression of EAAT2 in the hippocampus and cortex of WT and gp120 tgm mice (scale bar: 50 μm), and quantitative analysis using ImageJ software (ImageJ 1.54f). * *p* < 0.05, ** *p* < 0.01, and *** *p* < 0.001.

In vitro assays, we preliminarily demonstrated that KYNA promotes A2 astrocytogenesis and attenuates gp120-induced neurotoxicity by blocking the α7nAChR/NF-κB signaling axis. We evaluated the expression of the NF-κB subunit pp65 in mouse brain using immunofluorescence. As expected, intraperitoneal injection of KYN and PROB potently blocked NF-κB overactivation in the hippocampus and cortex of gp120 tgm mice (Figure 12A). Meanwhile, after intraperitoneal injection of KYN and PROB, the glutamate content in the cerebrospinal fluid of gp120 tgm mice was significantly reduced and approached normal physiological levels (WT mice) (Figure 12B). Finally, to further investigate glutamate toxicity, we assessed the content of EAAT2 in the hippocampus and cortex of mice by immunohistochemistry. intraperitoneal injection of KYN and PROB rescued the trend of decreased EAAT2 in the hippocampus and cortex of gp120 tgm mice (Figure 12C). Interestingly, both NF-κB subunit pp65 and glutamate levels were lower in the brains of gp120^+^/α7nAChR^−/−^ mice than in gp120 tgm mice. These data are consistent with our hypothesis that intraperitoneal injection of KYN and PROB protects gp120 tgm mice from glutamate toxicity by blocking α7nAChR/NF-κB signaling activation, which promotes A2 astrocyte formation.

### 2.7. Intraperitoneal Injection of KYN and PROB Improves Cognitive Function in gp120 tgm Mice

**Figure 13:**
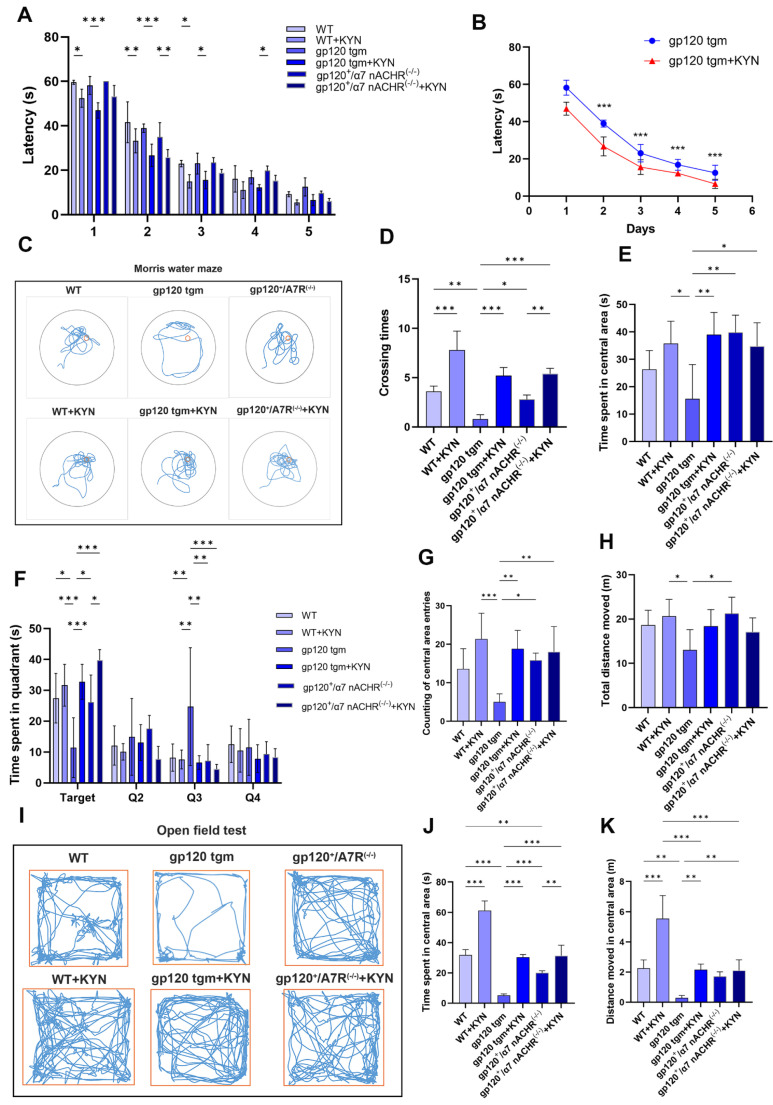
The effects of intraperitoneal injection of KYN and PROB on memory, cognition and mood in mice. (**A**) Latency of hidden platform training for 5 consecutive days in each group of mice. (**B**) Latency of hidden platform training for 5 consecutive days in gp120 tgm mice intraperitoneally injected with KYN and PROB and in gp120 tgm mice intraperitoneally injected with PBS. (**C**) Representative trajectories of mice in the spatial exploration test. (**D**) The number of times mice traversed the platform in the spatial exploration experiment. Time spent by mice exploring the center region (**E**) and four quadrants (**F**) of the water maze device. The number of times mice traversed the center region of the open field device (**G**), the time spent (**J**), and the distance moved (**K**). Representative trajectories (**I**) and total distance moved (**H**) by mice in the open field experiment. * *p* < 0.05, ** *p* < 0.01, and *** *p* < 0.001.

To determine whether the above neuropathological changes were reflected in cognitive deficits, we assessed spatial learning, memory capacity, and emotional abnormalities in mice using Morris water maze analysis and the open field test. The latency to find a hidden platform during five days of hidden platform training in the six groups of mice showed an overall gradual decrease, and the gp120 tgm mice required more time to find the platform than the WT mice (Figure 13A). More importantly, the latency of gp120 tgm mice injected with KYN was significantly shorter compared to gp120 tgm mice (Figure 13B), indicating that the learning ability of gp120 tgm mice was significantly improved. After hidden platform training, all mice were subjected to a spatial exploration test to assess memory. gp120^+^/α7nAChR^−/−^ mice had a significantly lower number of target platform crossings than WT mice and higher than gp120 tgm mice (Figure 13D). In addition, we observed that gp120 tgm mice spent significantly less time in the platform quadrant (Q1) than other mice. Notably, the target quadrant exploration time of gp120 tgm mice was significantly increased after injection of KYN or knockdown of α7nAChR (Figure 13F). Representative trajectories of mice in the water maze are shown in Figure 9C. The results in Figure 13C,E show that WT mice, gp120 tgm mice injected with KYN, and gp120^+^/α7nAChR^−/−^ mice performed intensive searches of the center where the platforms were located, while gp120 tgm mice had the worst centralization of search trajectories. In summary, KYN injection treatment significantly improved the learning and spatial memory abilities of gp120 tgm mice.

Next, we assessed the spontaneous exploration and anxiety-like behavior of mice in open fields. We found no difference in the total distance moved by gp120 tgm mice and WT mice (Figure 13H). However, the gp120 tgm mice group spent the least time in the center area and moved the shortest distance compared with the other groups (Figure 13J,K). Representative trajectories of mice in the open area are shown in Figure 13I. Notably, Figure 13G,I both indicate that gp120 tgm mice traversed the central area the least number of times and moved the shortest trajectories, suggesting a lack of exploration of the central area. In contrast, the gp120 tgm mice showed a significant improvement in their exploration behavior of the central area after KYN injection. Overall, these results showed that injection of KYN and PROB improved the exploratory behavior and anxiety of gp120 tgm mice.

## 3. Discussion

HIV-1 infection has been transformed into a chronic disease with the spread of combination antiretroviral therapy (cART) [35]. Because of poor BBB penetration of antiretroviral drugs and strong toxic side effects, there are already 19.5 million HAND patients in the world in 2023 [1,36,37]. Until now, there is no effective cure for HAND, so the increasing number of HAND patients and the aging of the population are worrisome. In our group’s previous study, we found that the HIV-1 envelope protein gp120 can induce microglia to release inflammatory factors and inflammatory vesicles NLRP3, which induces neuronal pyroptosis [4]. In addition, our latest study showed that gp120 was able to activate A1 astrocytes and induce neuroinflammation [6]. In the present study, we further found that KYNA was able to block α7nAChR/NF-κB signaling to promote A2 (neuroprotective) astrocyte generation, which enhances neurotrophic factor secretion and reduces glutamatergic toxicity, leading to amelioration of neurological damage and cognitive deficits in HAND. These findings are the beginning of our exploration in the complicated mechanisms of HAND neurodegenerative lesions, and provide a new clue for us to cure HAND.

Dysregulation of tryptophan metabolism has been associated with abnormal neuropsychiatric symptoms in HIV patients. HIV population studies have shown that the tryptophan-KYN pathway is dysregulated in HIV-1-infected individuals, with lower blood tryptophan concentrations and KYN/TRP ratios than uninfected individuals [38,39]. On the one hand, tryptophan inhibits HIV-1 viral replication and prevents viral entry into host cells [40]. On the other hand, KYN downstream metabolites may play opposite roles in the disease process, for example, KYNA and NAD^+^ may exert neuroprotective effects, whereas QUIN is positively correlated with depressive manifestations in patients and is neurotoxic [41]. The current study confirms that QUIN plays an important role in the progression of the HAND disease process. In vitro experiments have found that dendritic swelling, vacuolization, microtubule disruption, and cell death are observed in neurons chronically exposed to QUIN, and these manifestations are similar to the HAND neuropathology [42]. Through inhibition of QUIN production, the neurotoxicity and major depression phenotypes of HIV patients were ameliorated [43]. However, KYNA (another end product of KP) plays an important role in regulating neuroplasticity and cognition. KYNA has been shown to be neuroprotective under different experimental conditions of neurotoxicity [44], and intracerebral injection of KYNA at low doses effectively improves learning memory ability in experimental animals [42]. In particular, endogenous KYNA synthesized by glial cells is able to regulate the release of neurotransmitters such as dopaminergic and glutamatergic in the central nervous system by blocking α7nAChR and NMDA receptors, and thus it has unlimited potential for the treatment of neurodegenerative diseases [11,41]. Our latest study demonstrated that KYNA has the ability to inhibit A1 astrocytes and reduce the release of pro-inflammatory factors, which further confirms the neuroprotective effects of KYNA [6]. It is worth noting that our group previously designed animal experiments using high tryptophan dietary feeding that has been shown to have a proven safety profile [45]. However, during the metabolism of tryptophan to KYNA, it could not be determined that it was KYNA leading to the improvement of neurological function in mice due to the interference of the high number of tryptophan metabolites, the complex metabolic process of intestinal flora, and the difficulty of KYNA to cross the blood–brain barrier [46,47]. Because KYN can easily cross the blood–brain barrier and PROB can inhibit the loss of KYNA from the cerebrospinal fluid [48]. Therefore, based on the previous work of our team, the present study further determined the role of KYNA in neuropathological deficits and cognitive behavioral improvement in gp120 tgm mice by using an intraperitoneal injection regimen of KYN and PROB.

Astrocyte reactivity is a key player in all neurodegenerative pathologies [49], and different types of astrocytes are able to exert neuroprotective or neurotoxic effects in neurodegenerative diseases by regulating glutamate uptake, cytokine secretion and energy homeostasis [50]. Our latest study showed that KYNA inhibits A1 astrocyte activation and reduces pro-inflammatory cytokine secretion by blocking α7nAChR. Notably, in addition to A1 astrocytes (neurotoxic), another recognized reactive astrocyte is A2 (neuroprotective) [51]. Therefore, the present study investigated whether KYNA could inhibit A1 astrocytes while enhancing A2 astrocyte production, and the specific mechanism of their production. We found that gene expression specific for A2 astrocytes was significantly elevated in the brains of gp120^+^/α7nAChR^−/−^ mice compared to gp120 tgm mice. This result implies that blocking α7nAChR promotes A2 astrocyte formation. In previous studies by our group, *E. coli* K1 was able to promote bacterial meningitis by regulating NF-κB through α7nAChR [28,29], and this regulatory relationship was confirmed in other studies [52,53]. Interestingly, the NF-κB pathway is widely activated in neurodegenerative diseases and is able to activate astrocyte responsiveness [54,55]. In addition, the p65 gene, a subunit of NF-κB, is able to bind to the promoter sequence of EAAT2 in astrocytes, resulting in inhibition of EAAT2 expression, which leads to glutamate toxicity [30,31]. These findings imply that our α7nAChR/NF-κB signaling axis is closely related to A2 astrocytogenesis and its glutamate uptake capacity. Therefore, we utilized MLA, an inhibitor of α7nAChR, PNU-282987, an agonist, and Bay11-7085, an NF-κB inhibitor, to test our hypothesis, and the experimental results were inconsistent with our expectations. Taken together, we have reason to believe that KYNA can promote A2 astrocytogenesis and increase the EAAT2 expression level in A2 astrocytes by blocking the α7nAChR/NF-κB signaling axis, thereby attenuating gp120-induced glutamate toxicity.

KYN is a direct precursor of KYNA, QUIN and 3-hydroxykynurenine (3-HK), as well as other downstream metabolites of KP [56], so the limitation of the present study is that the effects of other KYN metabolites were not assessed in the mouse brain after intraperitoneal injection of KYN and PROB. It was shown that KYNA, 3-hydroxykynurenine (3-HK), QUIN and xanthurenic acid (XA) levels were significantly up-regulated in rat brain after supplementation with large amounts of exogenous KYN [57]. Therefore, the limitation of this study is that no measurements were made of the effects of intraperitoneal injection of KYN and PROB on the levels of other KP metabolites in the mouse brain. In addition, it was shown that KYN administration alone in the rat brain exhibited significantly higher levels of neurotoxic 3-HK than KYNA, and that excessive KYN loading resulted in memory impairment sequelae in immature healthy rats [56]. However, numerous studies have shown that KYN in combination with PROB is effective in reducing neurotoxicity by elevating KYNA levels [34,58,59]. Therefore, in the present study, the large loss of KYNA from the cerebrospinal fluid of gp120 tgm aged mice was avoided by the combination of PROB, which reversed the trend of KYNA levels in the brain being lower than 3-HK. Eventually the significant rise of KYNA in the brain significantly improved learning memory and cognitive impairment in gp120 tgm aged mice. Notably, low concentrations of KYNA (1–3 mM) do not exert an effect on α7nAChR [60]. Therefore, exploring reasonable combination concentrations of KYN and PROB is crucial for subsequent studies. In conclusion, KYN in combination with PROB may be more suitable for HAND clinical treatment, and further exploration of the metabolic processes of peripheral KYN and PROB in the brain and their influencing factors is necessary.

## 4. Materials and Methods

### 4.1. Isolation and Culture of Primary Astrocytes and Drug Treatment

The cerebral cortex was removed from anesthetized neonatal suckling rats (24 h) by aseptic manipulation, and the cerebrospinal membrane was isolated and placed in cold D-Hank’s solution according to our group’s preoperational manual. The cortex was clipped and digested by shaking with 0.25% trypsin (Gibco, Waltham, MA, USA) for 20 min at 37 °C. Tryptic digestion was stopped with a complete medium (10% FBS, 1% penicillin/streptomycin in a DMEM/F12 medium). The mixture was filtered through cell filters (75 μm pore size, Biosharp, He Fei, China) and inoculated into poly-d-lysine-coated T75 cell culture flasks (NEST, Wu Xi, China). After 10 days of culture, the cells were shaken in a shaking incubator (250 r/min) at 37 °C for 2 h and primary astrocytes were inoculated in 6-well plates at the desired density. Astrocytes with cell > 98% purity were identified by GFAP immunofluorescence staining (1:200 dilution, Proteintech, Rosemont, IL, USA, 16825-1-AP). Cultured astrocytes were treated with 0.01 μg/mL of HIV-1 gp120 [HIV-1/Clade B/C (CN54), China] for 12 h, followed by the administration of 25 μM KYNA (Sigma, St. Louis, MO, USA) for 12 h. In addition, astrocytes were pretreated with 10 nM methyllycaconitine (MLA, MCE, Monmouth Junction, NJ, USA), 500 nM PNU-120596 (MCE, NJ, USA) or 5 μM Bay11-7085 (GLPBIO, Montclair, CA, USA) for 1 h, respectively.

### 4.2. Quantitative Reverse Transcription PCR (RT-qPCR) Analysis

Total RNA was extracted from the samples using the RNAiso Plus reagent according to the manufacturer’s instruction manual. After identification of purity and concentration, reverse transcription was performed according to the protocol of the miRNA 1st Strand cDNA Synthesis Kit (by stem-loop) kit. Then, cDNA and target gene primers were run on a QuantStudio 6 Flex Real-Time Fluorescent Quantitative PCR System (Thermo Fisher, Waltham, MA, USA) using the Hieff^®^ qPCR SYBR Green Master Mix (Low Rox Plus) kit for quantitative PCR. Gene expression levels were measured based on the intensity of the detected fluorescence expression level.

### 4.3. Western Blotting

Samples were lysed with 100 μL of 1 × RIPA extraction buffer (Thermo Fisher, MA, USA). 4× SDS-PAGE upload buffer (Thermo Fisher, MA, USA) and cell lysate were mixed proportionally, and then the mixture was denatured at 100 °C for 10 min. Total proteins were separated using a 10% gradient SDS-PAGE gel, and then transferred to polyvinylidene difluoride membranes (PVDF membranes, 0.45 µm pore size, Sigma, MO, USA). The membranes were closed with Pierce™ Rapid Closure Buffer for 20 min and then incubated in primary antibody diluted in Universal Antibody Diluent (Sigma, MO, USA) for 10 h at 4 °C. Afterwards, the membranes were incubated with horseradish peroxidase (HRP)-coupled secondary antibody at room temperature for 1 h. The membranes were infiltrated using the Enhanced Chemiluminescence Kit (Bio-Rad Laboratories, 1705060, Hercules, CA, USA) for simultaneous visualization. The ImageJ software (ImageJ 1.54f) was used to quantify the gray scale of the bands.

Primary antibodies used were as follows: rabbit anti-GFAP (1:5000, 16825-1-AP), rabbit anti-C3/C3b/C3c (1:2000, 21337-1-AP), rabbit anti-CHRNA7 (1:2000, 21379-1-AP), rabbit anti-NF-κB p65 (1:2000, 80979-1-RR), rabbit anti EAAT2 (1:1000, 22515-1-AP), mouse monoclonal anti-GAPDH (1:5000, 17670-1-AP), goat anti-rabbit IgG-HRP (1:5000, PR30011), and goat anti- Mouse IgG-HRP (1:5000, PR30011). The above antibodies were purchased from Proteintech (IL, USA). Rabbit anti-Phospho-NF-κB p65 (Ser536) (1:2000, 80979-1-RR) was purchased from Cell Signaling Technology (Danvers, IL, USA).

### 4.4. Glutamate Uptake Assay

To assess the glutamate uptake capacity of astrocytes, glutamate content was measured using the glutamate assay kit (MAK004-1KT, Sigma, MO, USA). Explicit procedures for use are provided in the product manual. Replace the old medium with the DMEM/F12 medium containing 1 mM glutamate according to the published literature. After incubation in a 37 °C incubator for 40 min, 80 min and 120 min, 200 μL of the culture supernatant was collected and mixed with the reagents provided according to the manual. After 30 min in a 37 °C water bath, 100 μL of the mixed solution was taken and transferred to a 96-well cell culture plate. The absorbance of the product was measured at 450 nm using an enzyme meter. A standard sample was constructed in each assay using a medium containing a known concentration of glutamate.

### 4.5. Immunofluorescence

Treated cells were fixed with pre-cooled methanol for 10 min and permeabilized with 0.25% Triton X-100 for 10 min. The cells were then blocked with mixed buffer (1% *w/v* BSA, Sigma-Aldrich, USA; 22.52 mg/mL glycine in PBST) at room temperature for 1 h. Cells were incubated with rabbit anti-GFAP (1: 200, 16825-1-AP, Proteintech 16825-1-AP, Proteintech), rabbit anti-C3/C3b/C3c (1:200, 21337-1-AP, Proteintech), rabbit anti-EAAT2 (1:200, 22515-1-AP), and rabbit anti-Phospho-NF-κB p65 (Ser536) (1:200. 80979-1-RR, CST) were incubated at 4 °C for 12 h. Next, the samples were incubated with goat anti-rabbit IgG-HRP (1:200, PR30011, Proteintech) at room temperature and in the dark for 1 h. DAPI (4 μg/mL, Thermo Fisher, MA, USA, 62248) was used as a nucleus staining for 5 min. Finally, cells were observed for staining using a fluorescence microscope (E800 Nikon, Tokyo, Japan).

### 4.6. Animal Models and Treatments

The Medical Ethics Committee of Southern Medical University approved all animal experimental protocols (protocol number: L2018018). And we made every effort to minimize the number and suffering of the mice used. We purchased C57BL/6 mice (12 months old) from the Animal Experiment Center of Southern Medical University. gp120 tgm mice expressing gp120 LAV were used as described previously. The α7nAChR knockout (α7nAChR^−/−^) mice with a C57BL/6J background were obtained from Jackson Laboratories (B6.129S7-Chrna7tm1Bay/J, Stock No. 003232, Bar Harbor, ME, USA). The gp120 tgm mice were bred with α7nAChR^−/−^ mice to produce gp120^+^/α7nAChR^−/−^ mice. All animals with no specific pathogens were maintained on a 12 h light/dark cycle and had free access to food and water.

All mice were divided into 6 groups feeding 5 mice each, including WT + PBS, WT + KYN, gp120 tgm + PBS, gp120 tgm + KYN, gp120^+^/α7nAChR^−/−^ + PBS, gp120^+^/α7nAChR^−/−^ + KYN. Mice were injected intraperitoneally with PBS or KYN (400 mg/kg,) + probenecid (PROB, 200 mg/kg). The drug injection program was once every two days for 14 days. Mice were tested for spatial learning, memory, emotional and behavioral abnormalities using Morris water maze analysis and open field assay. Finally, mice were anesthetized by using tribromoethanol (T48402, Sigma, MO, USA) followed by taking cerebrospinal fluid, serum and brain. Half of the brains were fixed in 10% formaldehyde for immunofluorescence and immunohistochemical staining. The other part of the brain was rapidly frozen at −80 °C.

### 4.7. Behavioral Testing

The water labyrinth was a circular tank (1.2 m in diameter, divided into four quadrants called Q1, Q2, Q3 and Q4). The tank was filled with a 30 cm high white opaque liquid fill and maintained a water temperature of 24 °C. A hidden platform (2 cm below the water surface and 12 cm in diameter) was placed in the center of Q1. First, mice were trained for five consecutive days to find the platform hidden below the water surface by using the four markers surrounding the tank as spatial references. Mice was released into the water facing the edge of the tank from one of the four quadrants at a random location, and the four quadrants were used once a day. During a training session, mice were given 60 s to search for the platform; if the mice did not climb onto the platform, they were forcibly placed on the platform to rest for 30 s. Animal movements were tracked using a TSE VideoMot2 video tracking system (TSE Systems GmbH, Bad Homburg, Thuringia, Germany). After 5 consecutive days of hidden platform training, mice were given 60 s to search for the removed platform.

The open field device was a square box (40 × 40 × 30 cm). Mice were released into the center of the box and their behavior was recorded for 15 min via the TSE VideoMot2 video tracking system. The center area was a 20 × 20 cm square in the center of the box. The total distance moved, the distance moved in the center area, the time spent in the center area, and the number of entries into the center area were recorded.

### 4.8. Immunohistochemistry and Immunofluorescence

According to the Abcam immunohistochemistry (IHC) protocol, mouse hemi-brain samples were fixed, embedded and sectioned according to standard protocols. For antigen repair, sections were boiled with Tris-EDTA buffer (10 mM trimethylolaminomethane, 1 mM EDTA solution, 0.05% Tween 20, pH 9.0) for 20 min and rinsed under running water for 10 min. Sections were then blocked with 1% BSA and incubated with the following primary antibodies: rabbit anti-GFAP (1:400) and rabbit anti-EAAT2 (1: 400). Next sections were incubated with secondary antibodies and DAB (3,3-diaminobenzidine). After washing, cell nuclei were visualized using hematoxylin re-staining. The optical density of immunostained areas was quantified using ImageJ software (ImageJ 1.54f).

Brain tissue sections were blocked with 1% BSA and incubated overnight with the following primary antibodies diluted with 1% BSA: rabbit anti-pp65 (1:200), rabbit anti-MAP2 (1:200) and rabbit anti-NeuN (1:200). After washing the sections, they were incubated with secondary antibodies of appropriate species and fluorescence at room temperature for 1 h. Nuclei were visualized by TBST washing followed by DAPI re-staining for 5 min, and then covered by the addition of anti-quenching sealer (P36961, Thermo Fisher, USA). Finally, they were photographed by fluorescence microscopy (Nikon Eclipse: TE 2000-E, Tokyo, Japan).

### 4.9. Statistical Analysis

Graphs and statistical significance were generated by GraphPad Prism 9 software. Differences between the two groups were analyzed using an unpaired Student’s *t*-test (two-tailed). Between-group differences between multiple groups were analyzed using one-way ANOVA followed by Tukey post hoc tests. * *p* < 0.05, ** *p* < 0.01 and *** *p* < 0.001 represent statistical significance.

## 5. Conclusions

In conclusion, our vitro study revealed that KYNA blocks a7nAChR/NF-κB signaling activation to promote A2 astrocyte formation and ameliorates neuropathological deficits of HAND by attenuating glutamate toxicity and secreting neurotrophic factors. On the basis of our team’s previous study, we further clarified that endogenous KYNA is a neuroprotective factor for the treatment of HAND by intraperitoneal injection of KYN and PROB into gp120 tgm mice. This study provides new evidence that KYNA prevents and treats HAND

## Data Availability

Data are contained within the article.
